# Radioligand binding analysis of *α*_2_ adrenoceptors with [^11^C]yohimbine in brain in vivo: Extended Inhibition Plot correction for plasma protein binding

**DOI:** 10.1038/s41598-017-16020-1

**Published:** 2017-11-22

**Authors:** Jenny-Ann Phan, Anne M. Landau, Steen Jakobsen, Dean F. Wong, Albert Gjedde

**Affiliations:** 1 Department of Biomedicine, Aarhus University, Aarhus, 8000 Denmark; 2 Department of Nuclear Medicine and PET centre, Aarhus University Hospital, Aarhus, 8000 Denmark; 3 Department of Radiology & Radiological Science, Johns Hopkins University Hospital, Baltimore, 21231 USA; 4 Translational Neuropsychiatry Unit, Aarhus University Hospital, Aarhus, 8240 Denmark; 5 Radiology, Psychiatry, Neuroscience, Neurology, Environmental Health Sciences, Johns Hopkins University, Johns Hopkins Medical Institutions, JHOC Bldg room 3245, 601 N. Caroline St., Baltimore, MD 21287 USA; 6 Department of Neuroscience, University of Copenhagen, Copenhagen, 2200 Denmark; 7 Department of Neurology and Neurosurgery, McGill University, Montréal, Québec Canada; 8 Neurosciences Research Center, Tabriz University of Medical Science, Tabriz, Iran; 9 Department of Nuclear Medicine, Odense University Hospital, Odense, 5230 Denmark; 10 Department of Clinical Medicine, University of Southern Denmark, Odense, 5230 Denmark

## Abstract

We describe a novel method of kinetic analysis of radioligand binding to neuroreceptors in brain *in vivo*, here applied to noradrenaline receptors in rat brain. The method uses positron emission tomography (PET) of [^11^C]yohimbine binding in brain to quantify the density and affinity of *α*_2_ adrenoceptors under condition of changing radioligand binding to plasma proteins. We obtained dynamic PET recordings from brain of Spraque Dawley rats at baseline, followed by pharmacological challenge with unlabeled yohimbine (0.3 mg/kg). The challenge with unlabeled ligand failed to diminish radioligand accumulation in brain tissue, due to the blocking of radioligand binding to plasma proteins that elevated the free fractions of the radioligand in plasma. We devised a method that graphically resolved the masking of unlabeled ligand binding by the increase of radioligand free fractions in plasma. The Extended Inhibition Plot introduced here yielded an estimate of the volume of distribution of non-displaceable ligand in brain tissue that increased with the increase of the free fraction of the radioligand in plasma. The resulting binding potentials of the radioligand declined by 50–60% in the presence of unlabeled ligand. The kinetic unmasking of inhibited binding reflected in the increase of the reference volume of distribution yielded estimates of receptor saturation consistent with the binding of unlabeled ligand.

## Introduction

Deficient noradrenergic neurotransmission is implicated in a spectrum of brain disorders, including neurodegenerative and psychiatric disorders. Locus coeruleus is the major source of neuronal terminals engaged in noradrenaline synthesis, and pathological protein deposition together with loss of neurons at this site has been described in Alzheimer’s disease^1^. Similarly, attenuated levels of noradrenaline have been reported in Parkinson’s disease^2^. Attenuated noradrenergic activity is implicated in major depression, as indicated by findings of depleted noradrenaline in depressed patients with rapidly worsening symptoms^3^. Thus, multiple post-mortem studies have revealed elevated *α*_2_ adrenoceptor expression in suicide victims with a retrospective diagnosis of major depression, with upregulation of the *α*_2A_ adrenoceptor subtype noted particularly in the frontal and prefrontal cortices^4–6^.

Increased *α*_2_ adrenoceptor density is believed to compensate for attenuated noradrenaline release when experimental depletion of brain noradrenaline in rats led to upregulated cortical l *α*_2_ adrenoceptors^7^. In brains of patients who suffered from bipolar disorder, lower tyrosine hydroxylase immunoreactivity was found in locus coeruleus, consistent with deficient noradrenaline synthesis^8^. In support of low noradrenaline, a more recent PET study showed lower occupancy of noradrenaline transporters in locus coeruleus in patients with bipolar disorder or major depression, compared to healthy controls^9^. In attention-deficit/hyperactivity disorder (ADHD), impulsive behavior was associated directly with low noradrenergic tone^10^.

Central noradrenaline is known to modulate cognitive functions, such as arousal, mood, learning and memory (reviewed by Sara *et al*.^11^). To understand the neurobiology underlying brain disorders in the living brain, non-invasive PET using selective ligands can be used to monitor the activity of neurotransmission as a sign of disease severity and response to pharmacological therapy. The labeling of yohimbine with carbon-11 provided new opportunities to measure noradrenergic transmission in pathological conditions. [^11^C]yohimbine so far has been applied to image *α*_2_ adrenoceptor *in vivo* in different species, including humans^12^, pigs^13,14^, and rats^15–17^. However, the wide anatomical distribution of noradrenergic receptors complicates the quantification of receptor occupancy and receptor density.

The aims of the present study are twofold: First, we use yohimbine to obtain an estimate of the reference distribution volume of the radioligand in the absence of a true anatomical reference region. Second, we use kinetics to resolve the masking of ligand binding competition by pharmacological challenge that raises the plasma-free fraction of the radioligand.

Protein binding of drugs in plasma are of special interest in pharmacological studies because only the drug that is unbound to plasma proteins (i.e., is “free”) has access to target tissue and hence is biological active^18^. PET imaging of the brain with radiolabeleld ligands faces the same issue because the blood-brain barrier (BBB) permeability of the drug depends on the free-fraction of the ligand among many other factors. The CSF/plasma albumin ratio is 0.006, indicating that only a negligible fraction of albumin passes the BBB^19^. *In vitro*, the free drug concentration depends on factors such as protein quantity, drug affinity, and total drug concentration, while *in vivo*, factors such as metabolism, excretion and membrane transport, all influence the free drug concentration.

Quantification of neuroreceptor density with PET requires the determination of receptor availability at two or more different levels. The number of receptors available for radioligand binding can be modulated by agents that induce release or depletion of endogenous neurotransmitters, or by introduction of exogenous molecules that either bind to the receptors or affect the mechanism of binding of the endogenous ligands. Thus, pharmacological modulation changes the availability of sites for radioligand binding. Commonly, modulation of occupancy is achieved with unlabeled exogenous ligands that compete at the binding site. Receptor occupancy and density are then calculated from the fractional change of the receptor availability (also known as binding potential) after the pharmacologcial challenge.

To determine the binding potential, it is necessary to know the total volume of distribution of all labeled molecules in the tissue, *V*_T_, and the volume of distribution of molecules that cannot be displaced from binding (i.e., the volume of distribution indicative of “non-displaceable” binding), represented as *V*_ND_^20,21^. For radioligands that bind to some regions only (as in the case of dopaminergic radioligands), estimates of *V*_ND_ are the volumes of distribution in brain regions with no specific binding (reference regions). In contrast, for radioligands distributed in the entire brain, a reference volume must be estimated from regional values of *V*_T_ at two different different degrees of receptor occupancy, as described in Methods below.

Changes of free fractions of tracer in plasma, *f*_P_ , do not perturb the calculation of binding potentials in the presence of a non-binding reference region, because the value of *V*_ND_ is obtained from the degree of non-displaceble binding in the reference region, which scales with the freely distributed ligand in plasma. In the absence of a non-binding reference region, changes of *f*_P_ influence the magnitudes of *V*_T_ and *V*_ND_ estimates and hence may mask the competition from a blocking agent.

Here, we demonstrate a novel solution to issue of elevation of the magnitude of *f*_P_ in response to pharmacological challenge that may occur *in vivo*. The mathematical approach is designed to resolve the masking of competition attributable to the decreased protein binding of the tracer in plasma. As an example, we quantified the binding of [^11^C]yohimbine to widely distributed *α*_2_ adrenoceptors in the rat brain by an analysis that is not limited to *α*_2_ adrenoceptors but can be applied to radioligands of other receptors that lack a non-binding reference region.

## Results

### Tracer Accumulation as a Function of Time

**Figure 1:**
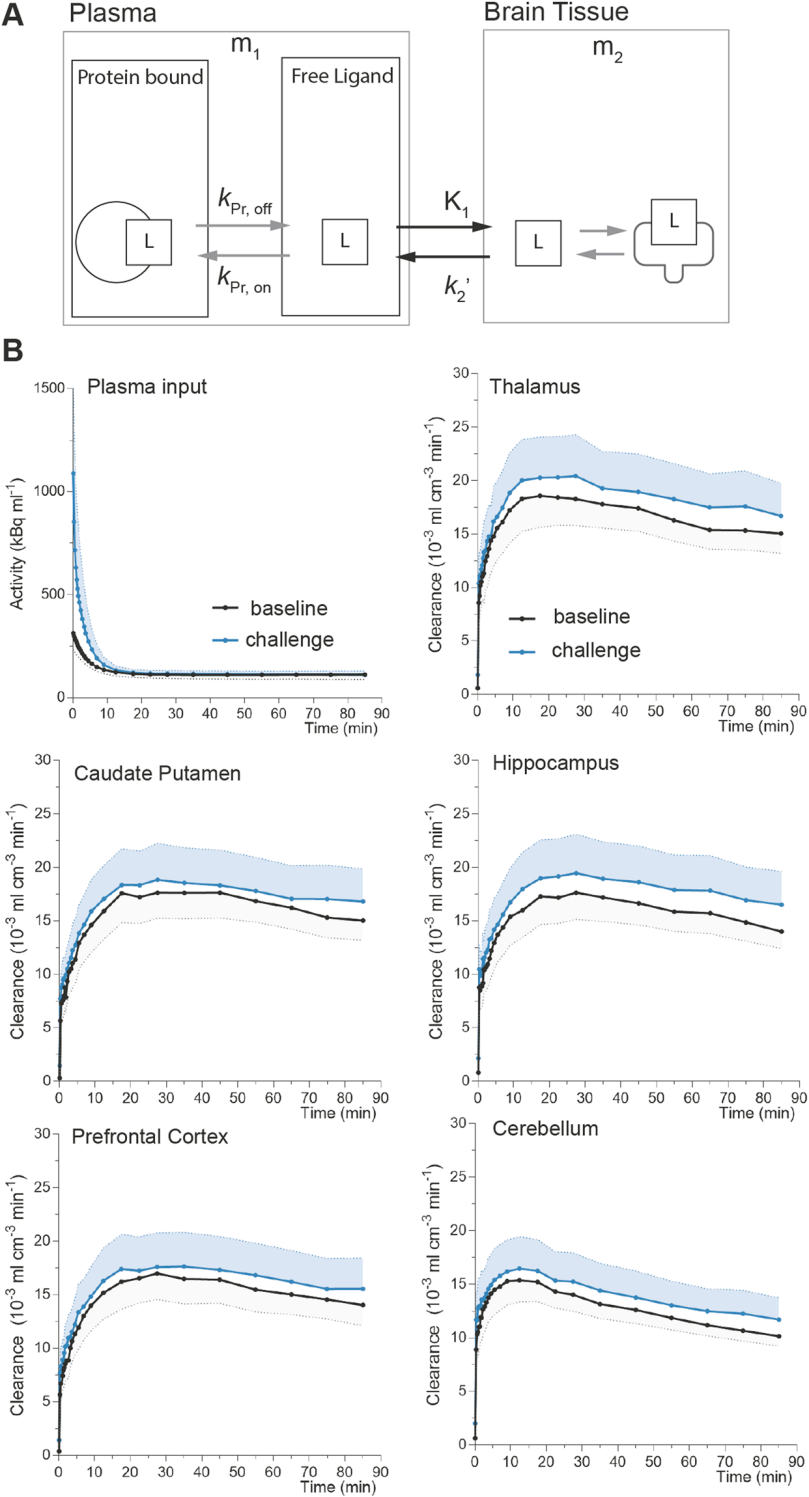
Tracer Accumulation as a Function of Time. Panel (A) illustrates the one-tissue compartment model used for data analysis. In this model, *K*_1_ represents the clearance from plasma to brain tissue while ${k}_{2}^{{\prime} }$ represents the fractional clearance of ligand from brain to plasma. Here, the free amount of ligand in plasma reflects the exchangeable compartment when only the unbound ligand crosses the BBB and enters brain tissue. The magnitude of *K*_1_ depends on the free concentration in plasma, cerebral blood flow, and the permeability surface area as well as the association and dissociation constants for plasma protein binding (depicted here as *k*_pr,on_ and *k*_pr,off_). The magnitude of ${k}_{2}^{{\prime} }$ depends on the concentration of ligand in brain tissue and the association and disassociation constants for receptor binding (shown as grey arrows on the right hand side). Initial evaluation of tracer accumulation in panel (B) demonstrates that average plasma input curves are of similar magnitude, indicating that the challenge condition did not significantly alter the baseline input function. The solid curves represent the average time-activities of individual curves divided by the integral of the respective plasma input to normalize for variations attributed to differences of injected radioactive dose, body weight and other bioavailability factors. Baseline is shown in black, yohimbine challenge in blue, and the shaded areas indicate the standard error of the mean. The time-activity curves (TACs) at challenge condition were markedly higher compared to baseline across all regions, suggesting that the inhibition effect is not immediate visible.

To evaluate the pattern of [^11^C]yohimbine binding to *α*_2_ adrenoceptors, we examined the binding at two levels of receptor occupancy, at baseline and at challenge with unlabeled yohimbine (0.3 mg/kg) administered as an i.v. bolus. The average time-activity curves (TACs) in plasma and brain regions show the kinetic behavior of [^11^C]yohimbine as it accumulates in the tissue (Fig. 1B). The clearance of the radioligand from the circulation occurred rapidly within the first 10 min after intravenous bolus injection. The uptake in cerebellum peaked at 10 min post-injection, while the accumulation peaked later in other brain regions at 20 min. TAC of the unlabeled yohimbine challenge revealed prominently elevated early uptake compared to baseline, showing that the effect of the challenge is not apparent exclusively from the TACs.

### Increased *K*_1_ May Indicate Displacement of Plasma Protein Binding

**Figure 2:**
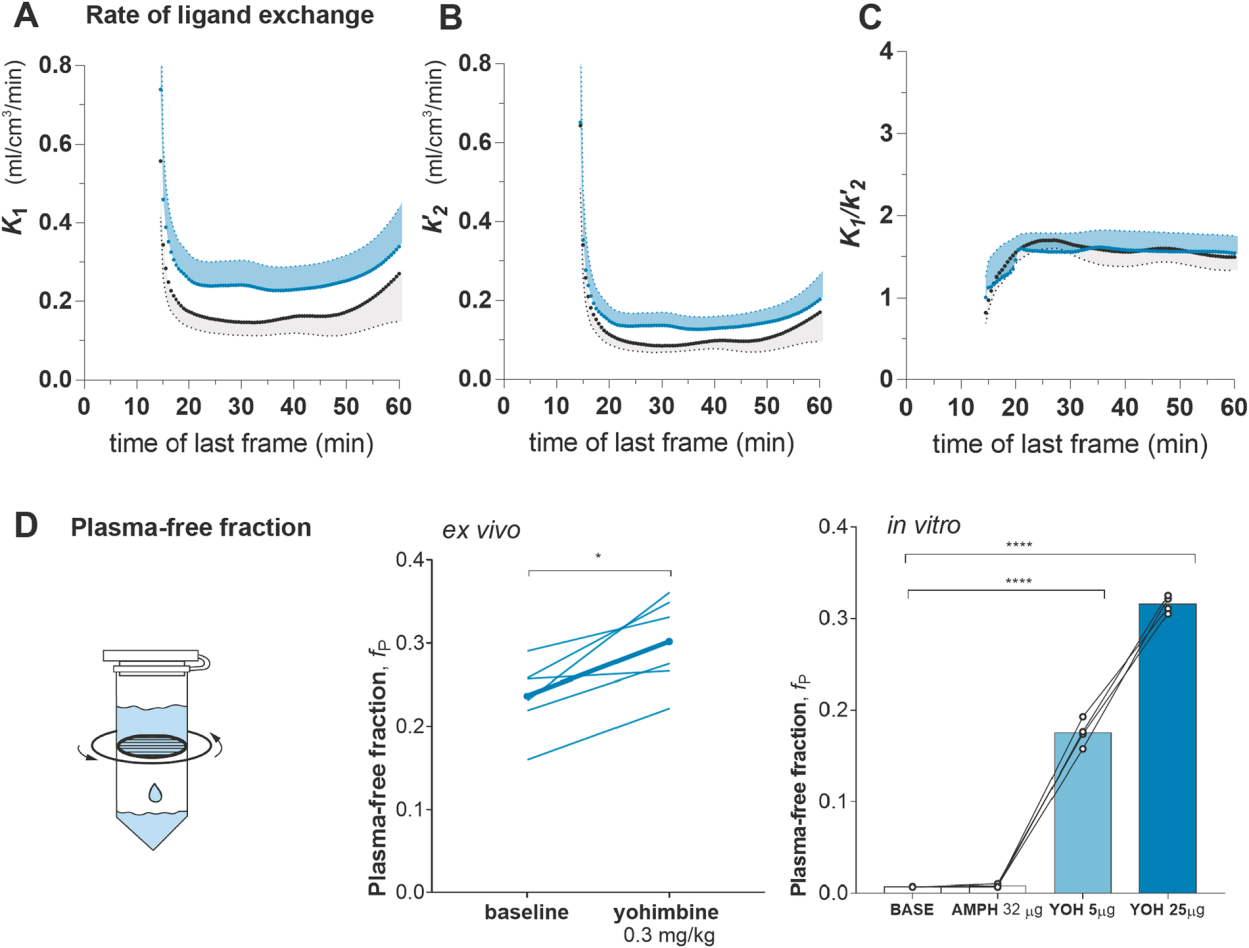
*K*_1_ and ${k}_{2}^{{\prime} }$ as Functions of Time and Plasma Free Fractions Determined in Separate Groups. The clearance from plasma to brain, *K*_1_ (panel (A)), and the fractional clearance from brain to plasma, ${k}_{2}^{{\prime} }$ (panel (B)), were calculated as functions of time using iterative analysis where each point represents a time duration of 14.5 minutes. Estimates of *K*_1_ and ${k}_{2}^{{\prime} }$ from were plotted against the time of the last frame of linearization, baseline in black,and yohimbine challenge in blue, with the shaded areas indicating the standard error of the mean. Both *K*_1_ and ${k}_{2}^{{\prime} }$ estimates were markedly higher in the challenge than in the baseline condition. Elevated ${k}_{2}^{{\prime} }$ in the challenge condition indicates displacement on *α*_2_ adrenoceptor sites due to competition with unlabeled yohimbine, whereas increased *K*_1_ suggest that more free ligand crosses BBB in the challenge condition due to displacement of plasma protein binding. Because of two simultaneous opposite effects, the ratio of *K*_1_/${k}_{2}^{{\prime} }$ at challenge condition (**C**) was at the same magnitude as baseline. This supports that yohimbine challenge was masked by elevated *f*_P_ . To confirm this notion, we measured *f*_P_ by means of plasma ultrafiltration (**D**) in a separate group of animals. In the *ex vivo* setup (D middle), 0.3 mg/kg yohimbine administered i.v. resulted in a significant increase of *f*_P_ by 30% as compared to baseline (P < 0.05). In the *in vitro* study, plasma was drawn before amphetamine or yohimbine were added to the samples. This showed that amphetamine did not change *f*_P_ , whereas yohimbine challenge produced a dose-dependent increase. Both low dose and high dose challenge increased *f*_P_ significantly as compared to baseline (****P < 0.0001). The thick line in D (middle) represents the group average, and the thin lines show estimates in individual animals, which connect estimates at baseline and challenge in the same animals.

As for the trends of TACs, the magnitude of unidirectional clearance from plasma to brain tissue, represented by *K*_1_, was markedly elevated in the challenge condition. The value of *K*_1_ at the challenge rose initially and maintained higher values during the time of acquisition than in the baseline (Fig. 2A). The magnitude of the fractional clearance from brain to plasma, symbolized by ${k}_{2}^{{\prime} }$, likewise was higher in the challenge than in the baseline condition, to the same relative extent as the value of *K*_1_ (Fig. 2B). The ratio of the the estimates of *K*_1_ and ${k}_{2}^{{\prime} }$ remained similar throughout the acquisition in both conditions (Fig. 2C). The increased values of *K*_1_ and ${k}_{2}^{{\prime} }$ in response to yohimbine challenge are the results of two opposite effects, one the blocking at *α* adrenoceptor sites by unlabeled yohimbine that underlies the elevated value of ${k}_{2}^{{\prime} }$, and the other the decreased protein binding reflected in the increased estimate of *f*_P_ that accounts for the elevated value of *K*_1_.

The early peak of *V*_T_ curves (Fig. 1C) together with the elevated *K*_1_ (Fig. 2A) suggest that the increased values of *f*_P_ reflect the effects of protein binding blocked by an unlabeled competitor. More than 80% of yohimbine in the circulation is bound to plasma proteins^22^ that renders [^11^C]yohimbine prone to self-displacement at plasma protein sites with increased value of *f*_P_ . The higher *f*_P_ of [^11^C]yohimbine in turn masks the competition from unlabeled yohimbine in the brain tissue.

To determine the degree of masking, we measured the free fractions of [^11^C]yohimbine at baseline and challenge conditions by means of plasma ultrafiltration in a separate group of animals. We determined the values of *f*_P_ by administration of 0.3 mg/kg yohimbine. The challenge with unlabeled ligand *ex vivo* significantly raised the value of *f*_P_ by 30% on average compared to baseline (P < 0.05) (Fig. 2D middle panel). To exclude the possibility that the elevation of *f*_P_ is due to competition from hepatic metabolism or renal elimination, we repeated the experiment *in vitro* (Fig. 2D right panel). *In vitro*, the experiments confirmed no change of the value of *f*_P_ with amphetamine challenge. As expected, the magnitude of *f*_P_ increased significantly (P < 0.001) in response to challenge with unlabeled yohimbine, in proportion to the yohimbine dose.

### Extended Inhibition Plots and Binding Potentials

On the basis of the substantial change of the value of *f*_P_ shown *ex vivo* and *in vitro*, we applied the Extended Inhibition Plot to resolve the volume of distribution of non-displaceable radioligand at inhibition, *V*_ND(i)_. In the analysis, the volume of distribution of non-displaceable radioligand at baseline, *V*_ND(b)_, is required to determine the magnitude of *V*_ND(i)_ at the challenge.

Previously, in the same strain of animals with same PET protocol, we confirmed that the values of *V*_ND(b)_ and *f*_P_ did not change when [^11^C]yohimbine binding was challenged with amphetamine (2 mg/kg)^16^, using the plasma input function. The plasma input was not metabolite corrected because data from two rats revealed no radioactive metabolites, as for radioactive metabolites in pigs^14^. Because the value of *f*_P_ remained the same with amphetamine challenge, we used the Inhibition Plot to obtain a value of *V*_ND(b)_ of 0.286 ml cm^−3^. The similar experiments and analyses of the previous study allowed us to apply the value of *V*_ND(b)_ of the previous study to the present study.

**Figure 3:**
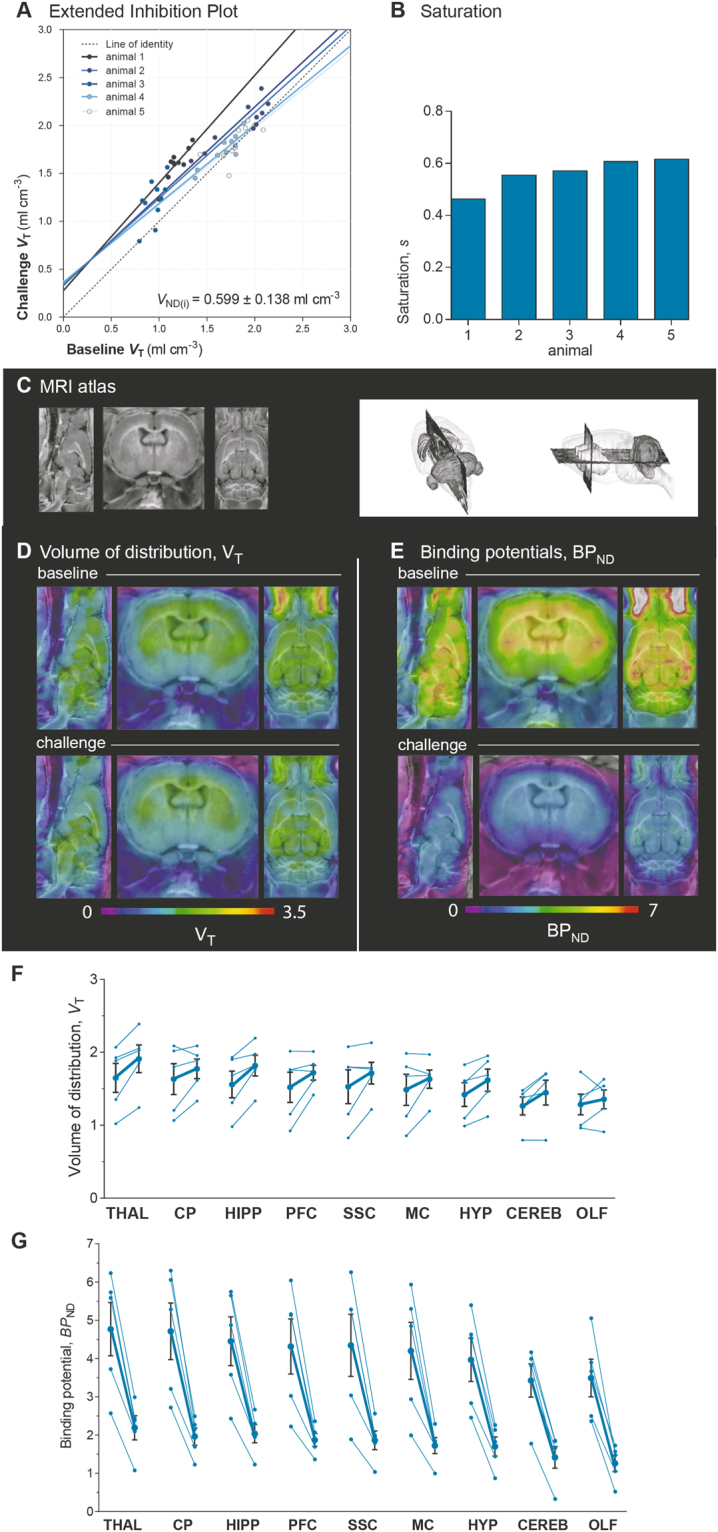
Extended Inhibition Plot. Due to significantly increased *f*_P_ in response to challenge, the Extended Inhibition Plot (eq. 36) (**A**) was applied to solve *V*_ND(i)_. In the Extended Inhibition Plot, regional estimates of *V*_T_ at challenge condition are plotted versus the estimates at baseline. *V*_ND(b)_ was set to 0.286 ml cm^−3^, because this value was previously assessed in an identical strain of rats^16^. The solid lines illustrate the regression in individual animals and the stippled line illustrates the line of identity. The plots were constrained to produce one population-wise *V*_ND(i)_ value, which yielded 0.599 ml cm^−3^. The saturation, *s*, obtained by the Extended Inhibition Plot is presented for individual animals in (**B**). This revealed that 0.3 mg/kg yohimbine challenge blocked in average 56% of the available receptors. Panel (C) shows the MRI atlas, which the parametric images were superimposed on. Parametric images of *V*_T_ in panel (D) show that yohimbine challenge did not change [^11^C]yohimbine *V*_T_ compared to baseline due to a masked effect. The real inhibition effect of yohimbine challenge was revealed when *BP*_ND_ was calculated using *V*_ND(i)_ at 0.599 ml cm^−3^ (panel (E)). Correspondingly, the quantification of *V*_T_ and *BP*_ND_ at steady-state in panels (F,G) are consistent with the parametric maps. The thin lines in (**F**) connect *V*_T_ at baseline and challenge in the same animal, and the thick line shows the average of the population. *V*_T_ was increased upon challenge with unlabelled yohimbine. The calculation of the *BP*_ND_ in (**G**) revealed a significant inhibition effect in response to challenge with unlabelled yohimbine (****P < 0.0001). The regional estimates of *V*_T_ and *BP*_ND_ are listed for individual animals in Table 1. THAL thalamus, CP caudate putamen, HIPP hippocampus, PFC prefrontal cortex, SSC somatosensory cortex, MC motor cortex, HYP hypothalamus, CEREB cerebellum, OLF olfactory bulb.

The graphical analysis yielded an estimate of *V*_ND(i)_ at 0.599 ml cm^−3^ (Fig. 3A), and the degree of saturation, *s*, revealed that 56% of available receptors were blocked by unlabeled yohimbine, as shown in Fig. 3B.

The quantification of *V*_T_ at the challenge condition revealed the masked effect reflected in the elevated value of *f*_P_ , as illustrated both by the parametric images (Fig. 3D) and by regional *V*_T_ estimates (Fig. 3F). To determine the real pharmacological displacement of [^11^C]yohimbine binding, we estimated the value of the binding potential, *BP*_ND_, from the estimates of *V*_T_ and the volumes of distribution of non-displaceable ligand, *V*_ND(i)_ and *V*_ND(b)_, respectively. As presented in Fig. 3E,G, calculations of *BP*_ND_ (by eq. 37 in Methods) revealed a significant decrease of *BP*_ND_ by the unlabeled ligand challenge. From the regionally differential estimates of *BP*_ND_, we found the highest *BP*_ND_ in thalamus, caudate putamen, hippocampus and cortical regions, with cerebellum and olfactory bulb at the lowest binding. The estimates of *BP*_ND_ decreased significantly by 50–60% on average in response to the unlabeled ligand challenge, with a consistent reduction in all animals. The estimates of *V*_T_ and *BP*_ND_ are listed in detail in Table 1.

**Table 1:** Volumes of Distribution and Binding Potentials.

A	Volumes of Distribution, *V*_T_														
	rat 1			rat 2			rat 3			rat 4			rat 5		
VOI	BASE	COLD	(%)	BASE	COLD	(%)	BASE	COLD	(%)	BASE	COLD	(%)	BASE	COLD	(%)
THAL	1.02	1.24	(22)	2.07	2.39	(15)	1.35	1.85	(37)	1.93	2.05	(7)	1.88	2.02	(7)
CP	1.06	1.33	(25)	2.02	2.09	(3)	1.20	1.61	(34)	2.09	1.96	(−6)	1.80	1.89	(5)
HIPP	0.98	1.33	(36)	1.93	2.19	(14)	1.31	1.76	(35)	1.90	1.97	(4)	1.68	1.82	(9)
PFC	0.92	1.41	(53)	2.01	2.01	(0)	1.15	1.61	(40)	1.76	1.74	(−1)	1.76	1.84	(4)
SSC	0.83	1.22	(47)	2.08	2.13	(3)	1.15	1.67	(45)	1.80	1.77	(−2)	1.80	1.79	(0)
MC	0.85	1.19	(39)	1.98	1.97	(−1)	1.12	1.62	(44)	1.67	1.68	(1)	1.80	1.70	(−6)
HYP	0.99	1.12	(13)	1.58	1.88	(18)	1.10	1.46	(33)	1.83	1.95	(7)	1.61	1.69	(5)
CEREB	0.79	0.79	(0)	1.48	1.71	(16)	1.26	1.59	(27)	1.43	1.70	(19)	1.38	1.45	(5)
OLF	0.96	0.91	(−6)	1.33	1.63	(22)	1.00	1.23	(23)	1.73	1.48	(−15)	1.40	1.54	(10)
**B**	**Binding potentials**, ***BP***_**ND**_														
	**rat 1**			**rat 2**			**rat 3**			**rat 4**			**rat 5**		
**VOI**	**BASE**	**COLD**	**(%)**	**BASE**	**COLD**	**(%)**	**BASE**	**COLD**	**(%)**	**BASE**	**COLD**	**(%)**	**BASE**	**COLD**	**(%)**
THAL	2.57	1.07	(−58)	6.23	2.99	(−52)	3.72	2.09	(−44)	5.73	2.43	(−58)	5.59	2.38	(−57)
CP	2.72	1.22	(−55)	6.06	2.49	(−59)	3.20	1.69	(−47)	6.30	2.26	(−64)	5.28	2.15	(−59)
HIP	2.43	1.22	(−50)	5.75	2.66	(−54)	3.57	1.95	(−46)	5.65	2.30	(−59)	4.87	2.05	(−58)
PFC	2.22	1.36	(−39)	6.04	2.36	(−61)	3.02	1.69	(−44)	5.16	1.90	(−63)	5.14	2.06	(−60)
SSC	1.88	1.03	(−45)	6.26	2.56	(−59)	3.03	1.79	(−41)	5.28	1.95	(−63)	5.28	1.99	(−62)
MC	1.99	0.99	(−50)	5.94	2.29	(−61)	2.93	1.71	(−42)	4.85	1.81	(−63)	5.30	1.84	(−65)
HYP	2.45	0.87	(−65)	4.53	2.13	(−53)	2.83	1.44	(−49)	5.40	2.26	(−58)	4.63	1.82	(−61)
CEREB	1.77	0.32	(−82)	4.16	1.85	(−56)	3.39	1.66	(−51)	3.99	1.84	(−54)	3.81	1.42	(−63)
OLF	2.36	0.51	(−78)	3.67	1.72	(−53)	2.50	1.05	(−58)	5.05	1.46	(−71)	3.90	1.57	(−60)

The magnitude of *V*_T_ (**A**) at challenge increased significantly compared to baseline (*P < 0.05). The correct *V*_ND(i)_ estimate obtained with the Extended Inhibition Plot was used to calculate *BP*_ND_ (**B**). This revealed the real inhibition effect of yohimbine challenge with 50–60% decline of *BP*_ND_ in the challenge condition compared to baseline (***P < 0.0001).

VOI volume of interest, BASE baseline, COLD challenge with unlabeled yohimbine, % percent-wise change. THAL thalamus, CP caudate putamen, HIPP hippocampus,PFC prefrontal cortex, SSC somatosensory cortex, MC motor cortex, HYP hypothalamus, CEREB cerebellum, OLF olfactory bulb.

### Receptor Density

**Figure 4:**
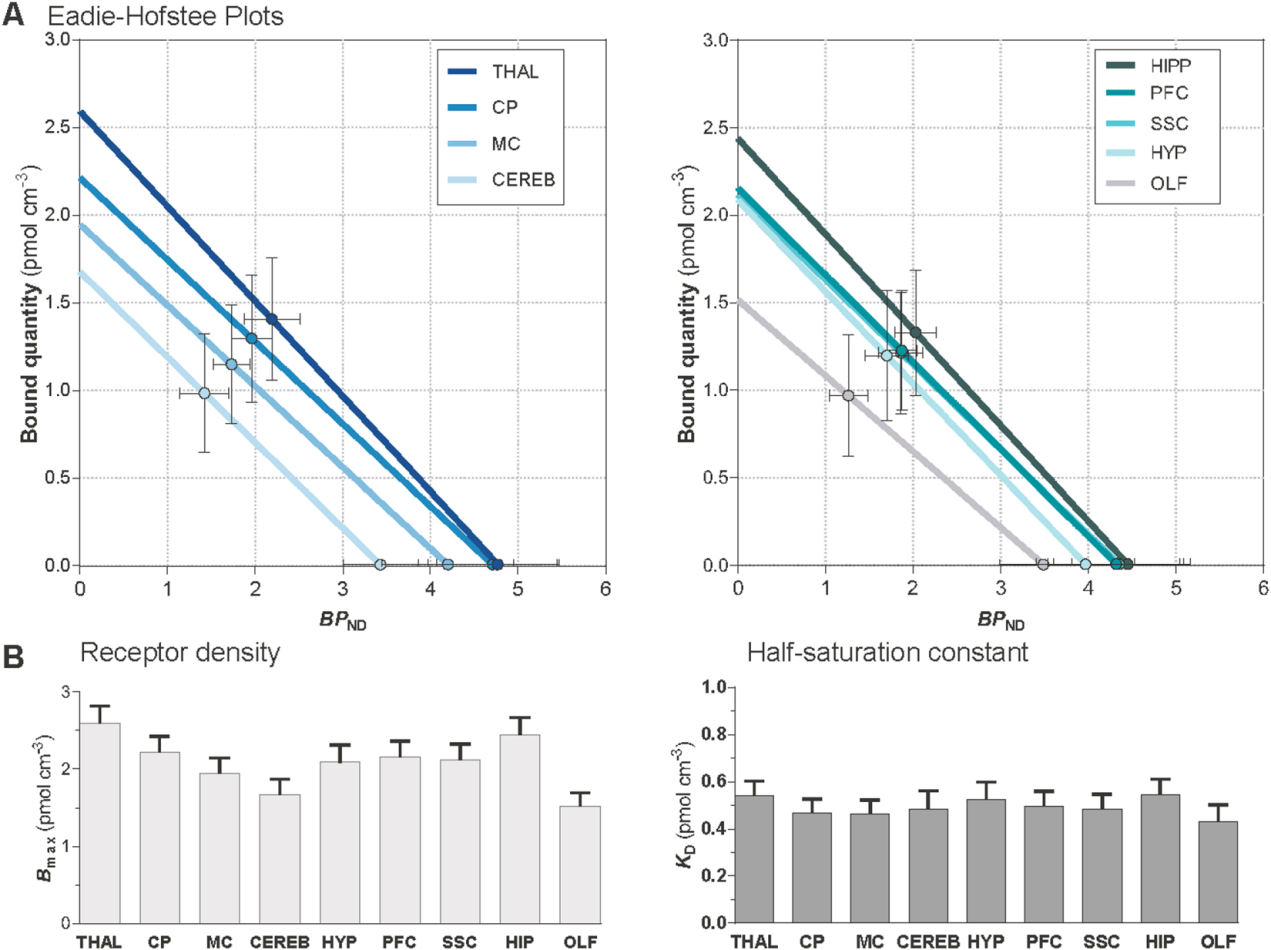
Eadie-Hofstee Plot. Eadie-Hofstee Plot (eq. 1 in Methods) presented in (**A**) solves the receptor density (*B*_max_) and affinity (1/*K*_D_) from a linear relationship between the bound quantity and binding potentials. The bound quantity was calculated using specific activity and *BP*_ND_ (eq. 2 in Methods). Receptor densities was obtained from the Y-intercept and the half-saturation constant, *K*_D_, as the slope. Each bar represents the mean, the error bars correspond to the S.E.M. The highest densities of *α*_2_ adrenoceptors were found in thalamus, hippocampus, caudate putamen and prefrontal cortex, whereas lowest densities were found in cerebellum and olfactory bulb ((**B**) left). *K*_D_ was at a similiar level in all examined regions at approximately 0.5 mM ((**B**) right) The regional receptor densities and *K*_D_ are also listed in Table 2, and the specific activities that were used for calculation of bound quantity are listed in detail in Table 3. THAL thalamus, CP caudate putamen, HIPP hippocampus, PFC prefrontal cortex, SSC somatosensory cortex, MC motor cortex, HYP hypothalamus, CEREB cerebellum, OLF olfactory bulb.

The values of *BP*_ND_ enabled us to determine the *α*_2_ adrenoceptor density *in vivo*. As presented in Fig. 4A, we estimated values of receptor density, *B*_max_, and receptor affinity (1/*K*_*D*_) by means of Eadie-Hofstee plots. We calculated the bound quantity according to eq. 2, based on the information of the *BP*_ND_ and specific activity. We found the greatest receptor densities in thalamus, caudate putamen, and cortical areas, with the lowest densities in cerebellum and olfactory bulb as presented in Fig. 4B. The regional receptor densities are listed in detail in Table 2 and specific activity in Table 3.

**Table 2:** Receptor Density and Half-Saturation Constant.

	*B*_max_ (pmol cm^−3^) mean ± S.E.M.	*K*_D_ (nM) mean ± S.E.M.
THAL	2.59 ± 0.50	0.54 ± 0.14
CP	2.21 ± 0.48	0.47 ± 0.13
HIPP	2.44 ± 0.51	0.55 ± 0.15
PFC	2.16 ± 0.46	0.50 ± 0.14
SSC	2.12 ± 0.47	0.48 ± 0.14
HYPP	2.09 ± 0.50	0.52 ± 0.16
MC	1.95 ± 0.44	0.46 ± 0.14
CEREB	1.68 ± 0.45	0.49 ± 0.17
OLF	1.52 ± 0.41	0.43 ± 0.16

The receptor density (*B*_max_) and affinity (1/*K*_D_), solved from Eadie-Hofstee plot (shown in Fig. 4A), are listed here for individual animals. This demonstrates that areas with densely expressed *α*_2_ adrenoceptors include thalamus, hippocampus, caudate putamen, and prefrontal cortex, whereas cerebellum and olfactory bulb were regions with lower density. Interestingly, *K*_D_ was higher in regions with enriched *α*_2_ adrenoceptors and low in regions with low density, supporting that receptor affinity is greater in areas with low receptor expression.

**Table 3:** Specific Activity.

	Radioactivity (MBq)		Total quantity (pmol)		Specific activity (kBq pmol^−1^)	
	BASE	COLD	BASE	COLD	BASE	COLD
rat 1	16.2	20.5	0.164	243	98988	84
rat 2	21.5	25.5	4.374	242	4916	106
rat 3	30.7	31.1	0.847	224	36267	139
rat 4	30.2	39.1	0.564	234	53514	167
rat 5	27.4	32.6	0.049	240	554889	136
mean	25.35	28.6	1.019	235	161910	122

The specific activity was used to quantify the bound quantity from eq. 2. The table shows the injected radioactivity, total quantity of unlabeled yohimbine and specific activity for individual animals at baseline and challenge condition.

BASE baseline, COLD challenge with unlabeled yohimbine.

## Discussion

We characterized the binding profiles of [^11^C]yohimbine to *α*_2_ adrenoceptors in rat brain, using non-invasive PET. We quantified the binding potentials and regional receptor densities by means of competition analysis. The results of the study demonstrate a significant increase in *f*_P_ in response to challenge with unlabeled yohimbine at the dose of 0.3 mg/kg. We argue that the elevation of *f*_P_ was caused by displacement of radioligand from plasma proteins, which ultimately masked the effect of the pharmacological inhibition in the brain. The masked effect is evident from estimates of *V*_T_ that reflect the total concentration in brain relative to the concentration in plasma. Furthermore, the challenge condition displayed consistently higher values of *K*_1_ in comparison to baseline, supporting the attribution of greater clearance of [^11^C]yohimbine and increased magnitude of *f*_P_ due to decreased protein binding. When the magnitude of *K*_1_ is influenced by specific biological variables, including BBB permeability, cerebral blood flow (CBF) and *f*_P_ , it is important to consider the changes of CBF a potential confound. In the challenge condition, the magnitude of *K*_1_ may be influenced by pharmacological action of yohimbine in the cardiovascular system. In humans and pigs, yohimbine is known to reduce CBF and increase blood pressure and heart rate^14,23^. Jakobsen *et al*.^14^ showed that CBF was unaffected by a low dose of yohimbine (0.07 mg/kg), while the higher dose (1.6 mg/kg) globally lowered CBF by 30%. In contrast, challenge with RX821002 had the opposite effect on CBF. In rats, yohimbine is known to raise blood pressure and heart rate^24^, while the effect on CBF is unknown.

The estimate of *K*_1_ was 0.2 ml/cm^3^/min, corresponding to a rate of escape of 0.167 s^−1^ for a vascular volume of 0.02 of 0.02 ml/cm^3^ ^25^. Because there are two connected processes (escape from plasma protein binding and exchange across BBB), it is important to note that blood-brain transfer can be affected by the dynamics of protein binding (here symbolized by *k*_Pr,on_ and *k*_Pr,off_). When the rate of relaxation from the plasma protein binding is greater than the exchange of tracer across the BBB, the loss of ligand bound to plasma proteins is likely to keep pace with the transfer of the tracer to brain tissue. If so, the transfer across BBB must depend on the original fraction of unbound ligand in plasma, including the unlabeled challenger. The kinetics of yohimbine relaxation from albumin is not known, but the relaxation of bilirubin from plasma proteins was estimated to occur at the rate of 0.6 s^−1^ ^26^. If we assume that yohimbine leaves plasma protein binding at a rate similar to that of bilirubin, the free and bound yohimbine pools in brain vasculature are likely to maintain a constant ratio.

Another consideration is the perturbation of hepatic enzymatic clearance of [^11^C]yohimbine in the presence of unlabeled yohimbine at the challenge dose. However, inhibited elimination by peripheral organs is an unlikely explanation for the rise of the value of *f*_P_ , when the *in vitro* assay eliminates effects of peripheral organs. The widespread expression of *α*_2_ adrenoceptors in the periphery may be another contributor to elevation of *f*_P_ by displacement of radioligand from sites in heart, pancreas^27^, smooth muscles in blood vessels^28,29^, kidneys^30^, and platelets^31^.

Elevated *f*_P_ may have influenced the findings by Jakobsen and colleagues^14^ of a lack of dose-dependent reduction of labeled yohimbine accumulation with unlabeled yohimbine challenge in pigs. Competition with a low dose (0.07 mg/kg) yohimbine reduced the value of *V*_T_ of [^11^C]yohimbine in porcine brain by approximately 30%. However, pre-treatment with a 24-fold higher dose (1.7 mg/kg) failed to decrease *V*_T_ further. Another selective ligand of *α*_2_ adrenoceptors, RX821002, was used to challenge [^11^C]yohimbine binding in porcine brain, where the low dose of 0.15 mg/kg caused greater decline of the value of *V*_T_ than the higher dose of 0.7 mg/kg. Although values of *f*_P_ were not determined in the study by Jakobsen and colleagues^14^, increase of the value of *f*_P_ is a likely explanation, evident from findings *in vitro* that binding of yohimbine engages in dose-dependent competition with tritium-labeled yohimbine^32^ and with RX821002^33^.

In similar experiments by Smith *et al*.^34^, [^11^C]mirtazepin binding in porcine brain was sensitive to yohimbine competition in a dose-dependent manner, where challenge with 0.3 and 3.0 mg/kg yohimbine reduced [^11^C]mirtazepin binding by 23% and 43%, respectively. In contrast, challenge with RX821002 at dose of 0.1 mg/kg reduced [^11^C]mirtazepin binding by 35%, while a dose of 1 mg/kg failed to inhibit the binding further. It is unknown if the RX821002 challenge displaced plasma protein binding, but it is likely that RX821002 displaces binding to *α*_2_ adrenoceptor sites in peripheral organs that potentially contributes to a higher value of *f*_P_ .

Pharmacological challenge by means of PET is a common test of the reversibility of radioligand binding. However, a number of studies have reported unaltered or increased occupancy of receptors after pharmacological challenge: A study of baboon brains showed that occupancy of 5-HT_2A_ receptors by [^18^F]altanserin, administered as constant infusion, was unaltered in the presence of extracellular levels of serotonin, elevated with fenfluramine, a selective serotonin reuptake inhibitor^35^. The binding of [^18^F]altanserin is known to be sensitive to challenge with the 5-HT_2A_ receptor antagonist, SR 46349 in baboons^35^ and also to non-radiolabeled altanserin in rodent brain^36^. Similarly, a PET study of neuroinflammation revealed increased [^11^C] PBR28 binding to the translocator protein (TSPO) in baboon brain in response to i.v. injection of 0.1 mg/kg LPS (lipopolysaccharide)^37^. The increases of binding at 1 and 4 hours post-administration were accompanied by increase of pro-inflammatory cytokines, causing the authors to propose that systemic inflammation by LPS can lead to increased TPSO expression. However, studies with infrared spectroscopy have shown that LPS binds to plasma albumin with the ratio 10:1, which changed the secondary structure of albumin^38^. Therefore, it cannot be excluded that increased [^11^C] PBR28 binding after LPS administration is confounded by increase of *f*_P_ due to structural changes of albumin exerted by LPS. This possibility was confirmed recently by Hilmer *et al*.^39^ who showed that a low dose of LPS (0.1 ng/kg) administered i.v. to rhesus macaque monkeys increased [^11^C] PBR28 *f*_P_ by 38%, as *V*_T_ increased by 39%. Increased *f*_P_ was also found to be elevated in response to pharmacological challenge in a study of *α*_7_ nicotinic acetylcholine receptors with [^18^F] DBT-10^40^, where the challenge with a dose of the antagonist ASEM at 1.24 mg/kg produced a two-fold increase of *f*_P_. The authors quantified *V*_T_/*f*_P_ as an outcome parameter in order to determine the net effect of pharmacological blocking. Taken together, the examples illustrate that changes of *f*_P_ are held to occur commonly in PET studies and knowledge of changes of *f*_P_ will be necessary to determine the true effect of pharmacological challenge. Therefore, lack of inhibition or evidence of increased binding in future studies must raise the concern of changes to *f*_P_ , measured with plasma ultrafiltration or equilibrium dialysis.

The direct relationship between increased *f*_P_ and masked effects of challenge on *V*_T_ must be interpreted with caution when *f*_P_ and *V*_T_ are determined in separate groups of animals, as in the present study. Another limitation of the Extended Inhibition Plot is the source of the baseline value of *V*_ND_ (estimated in a condition where *f*_P_ remains constant). Ideally, estimation of two *V*_ND_ values requires at least three PET acquisitions. Instead, it may be possible to use a population-based value of *V*_ND(b)_ to estimate *V*_ND(i)_, as demonstrated in the present study. This approach requires homogeneity of the study populations, with the added limitation that biological variability affects the accuracy of the *V*_ND_ estimates. We note that the value of *f*_P_ determined by plasma ultrafiltration is a steady-state estimate, whereas the exchange *in vivo* is a dynamic process, as discussed above, making it difficult to numerically extrapolate changes in *f*_P_ to changes in *V*_T_. We suggest that *f*_P_ should be quantified solely to determine whether criteria are met to employ the original plot, without the extension. If there is significant change of the magnitude of *f*_P_ , the criteria may be met for the application of the Extended version of Inhibition Plot. As the plot operates with steady-state variables, the dynamic origin of *f*_P_ will not influence the outcome.

The findings of the present study show that the *α*_2_ adrenoceptors are distributed throughout the brain. Regions with the greatest *B*_max_ estimates included thalamus, hippocampus, and caudate-putamen, where the densities ranged between 2.4 and 2.5 pmol/cm^3^. The receptor density of caudate-putamen at 2.2 pmol/cm^3^ (corresponds to 220 fmol/mg protein) agrees with *in vitro* receptor binding results from rat caudate-putamen with [^3^H]rauwolscine, a stereoisomer of yohimbine that yielded an estimate of 197 fmol/mg protein^41^.

To enable comparison with other studies, we converted the values of *B*_max_ per unit wet weight to values per unit dry weight of protein. Assuming that brain tissue contains 10% protein and dry weight therefore is one tenth of wet weight^42^, the range of values of *B*_max_ in prefrontal cortex in this study is 216 fmol/mg protein, and the value of *K*_D_ is 0.5 nM. To compare, we listed values of *B*_max_ and *K*_D_ from cortical areas of rat brain in Table 4. The table shows that comparison with results of *in vitro* binding assays is not simple, because of the great variability of findings of *B*_max_ and *K*_D_ among *in vitro* studies, depending on the method used.

**Table 4:** Comparison of *B*_max_ and *K*_D_ estimates.

Reference	Methods					Results		
	Radioligand	pH	temp (°C)	mg^2+^	Blocking of NS	Brain area	*B*_max_ (fmol/mg protein)	*K*_D_ (nM)
This study	[^11^C]yohimbine	*in vivo*, physiological condition			mathematical, 0.3 mg/kg YOH	prefrontal cortex	216	0.50
Rout *et al*.^32^	[^3^H]yohimbine membrane assay	7.0	25.0	0 mM	noradrenaline 100 *μ*M	cortex	260	9.86
		7.0	25.0	10 mM	noradrenaline 100 *μ*M	cortex	157	11.06
Brown *et al*.^44^	[^3^H]yohimbine membrane assay	7.4	25.0	0	phentolamine 10 *μ*M	cerebral cortex	121	5.20
Brown *et al*.^45^	[^3^H]yohimbine membrane assay	7.4	25.0	0	phentolamine 10 *μ*M	cerebral cortex	121	5.30
Ribas *et al*.^7^	[^3^H]RX821002 binding assay	7.5	25.0	1 mM	adrenaline 10 *μ*M	parieto-occipital cortex	92.0	0.57
Ribas *et al*.^68^	[^3^H]RX821002 binding assay	7.5	25.0	1 mM	adrenaline 10 *μ*M	parieto-occipital cortex	81.0	0.50
Boyajian *et al*.^41^	[^3^H] -rauwolscine autoradiography	7.7	22	0	phentolamine 10 *μ*M	anterior forebrain	109	0.79

The estimates from the present study was compared with previous data from *in vitro studies*. The data and experimental conditions are listed here.

The values of *B*_max_ in prefrontal cortex in the present study are closest to the result of [^3^H]yohimbine binding assays by Rout *et al*.^32^ at 260 fmol/mg protein in the condition of no added magnesium in the assay. The authors demonstrated that [^3^H]yohimbine binding depends on many physiological factors such as pH, temperature, and Mg^2+^ concentration. We argue that the estimate by Rout *et al*.^32^ in absence of Mg^2+^ is closer to the physiological condition and therefore more directly comparable to the present results than the condition with 10 mM Mg^2+^ present, considering the physiological level of Mg^2+^ in brain tissue of 230 *μ*M^43^. While the *B*_max_ values are in range, the estimates of *K*_D_ are 20-fold higher than the present estimates. The discrepant values of *K*_D_ may relate to the method used to block non-specific binding. In the present study, the non-specific binding is computed as reflected in the value of *V*_ND_, while the concentrations of competitors delivered to the binding sites *in vitro* often are in doubt because of the non-physiological delivery necessitated by the *in vitro* condition. Rout and colleagues used 100 *μ*M noradrenaline to block non-specific binding, which may lead to an overestimation of *K*_D_ because noradrenaline and [^3^H]yohimbine compete at the specific binding sites of *α*_2_ adrenoceptor.

In comparison to the results from [^3^H]yohimbine membrane binding studies by Brown *et al*.^44,45^, values of *B*_max_ in the present study are 1.5-fold higher, while values of *K*_D_ are 10-fold lower. The variability of estimates of *B*_max_ and *K*_D_ therefore may be attributed by differences in the methods of blocking neccesary to determine non-specific binding. Brown and colleagues used 10 *μ*M phentolamine that is known to antagonize *α*_1_ and *α*_2_ adrenoceptors with higher selectivity to *α*_1_ adrenoceptor^46^. Thus, due to the affinity to *α*_1_ and *α*_2_ adrenoceptor, use of phentolamine may underestimate values of *B*_max_ and overestimate values of *K*_D_, as *α*_1_ and some *α*_2_ adrenergic sites both may be blocked. The same blocking method was applied in the study by Boyajian *et al*.^41^ to block [^3^H]RX821002 binding to *α*_2_ adrenoceptor, the results of which are in agreement with the findings of Ribas and colleagues^7^ with estimates of [^3^H]RX821002 *B*_max_ and *K*_D_ in the same range. Estimates of *B*_max_ generally are higher when determined with [^11^C]yohimbine than with [^3^H]RX821002, possibly due to differential affinities to *α*_2_ adrenoceptor subtypes. Yohimbine does not distinguish between the subtypes, whereas RX8201002 has a higher affinity to *α*_2*A*_ and *α*_2*C*_ than to *α*_2*B*_^33^. Therefore, use of RX8201002 may underestimate the desnity of *α*_2_ adrenoceptor sites. Altogether, more *in vivo* studies are essential to examine *α*_2_ adrenoceptors in health and disease when physiological variables strongly influence *α*_2_ adrenoceptor binding and are difficult to reproduce *in vitro*.

We estimated values of *B*_max_ and *K*_D_ with the Eadie-Hofstee plot from only two magnitudes of receptor occupancy, which may lower the accuracy. Ideally, more levels of occupancy yield more accurate regressions. However, due to the limited blood volume in rats, we did not perform additional PET acquisitions that would result in greater loss of blood that in turn would initiate compensatory physiological changes (increased heart rate and vasoconstriction) that affect radioligand delivery to the brain. The method presented here can be applied to larger mammals with greater blood volumes that enable estimates of *B*_max_ and *K*_D_ with higher accuracy. We acknowledge that receptor density and affinity estimated *in vivo* may not necessary reflect the ultimate estimates determined *in vitro*, because *in vivo* experiments often are performed in animals anesthetized with gas anesthetics known to elevate noradrenaline^47^.

In conclusion, we demonstrated the use of the Extended Inhibition Plot to quantify the binding potentials of [^11^C]yohimbine and density of *α*_2_ adrenoceptors in the living brain with PET. The method enables the analysis of future investigations of the noradrenergic and other transmission systems with wide distributions in brain, such as the nicotinic cholinergic system^48^.

## Methods

### Novel Quantitative Analysis of Specific Binding to Brain Receptors

We derived the present method from well-known principles of kinetics. We identified the following six steps to the determination of binding parameters of radioligands that achieve receptor occupancy. The binding parameters are constants in the fundamental equation that underlies the Eadie-Hofstee plot^49,50^,

1 $$B={B}_{{\rm{\max }}}-{K}_{{\rm{D}}}\,B{P}_{{\rm{ND}}}$$

where *B*_max_ is the maximum binding capacity and 1/*K*_D_ the affinity of the receptors. The variable *B* is the bound quantity of the ligand, and *BP*_ND_ is the binding potential of the radioligand at the specific degree of occupancy. To solve the equation of the Eadie-Hofstee plot for the two parameters, it is necessary to know the bound quantities of the ligand and the binding potentials in at least two conditions of different occupancy. The bound quantity is related to the binding potential by the relationship,

2 $$B=M\,(\frac{B{P}_{{\rm{ND}}}}{1+B{P}_{{\rm{ND}}}}),$$

where *M* is the total mass of the ligand per unit volume of brain tissue, calculated from the known specific activity of the radioligand. The binding potential in turn is the ratio between the quantities of specifically (and hence displaceably) bound ligand and the unbound (and hence non-displaceable) ligand, equal to the ratio of the volumes of distribution of displaceable and non-displaceable ligand quantities,

3 $$B{P}_{{\rm{ND}}}=\frac{{V}_{{\rm{T}}}-{V}_{{\rm{ND}}}}{{V}_{{\rm{ND}}}}.$$

where *V*_T_ is the apparent volume of distribution of all tracer in the tissue, and *V*_ND_ is the apparent volume of distribution of unbound and hence non-displaceable tracer in the tissue, relative to the concentration in arterial plasma at steady-state.

#### Integral uptake equation

The first step is the kinetic analysis of tracer accumulation. The simplest mathematical description of the pharmacokinetic behavior of radiotracers in brain is the one-tissue compartment model (Fig. 1A) that assumes approach to a secular steady-state across the blood-brain barrier,

4 $$\frac{d{m}_{1}(t)}{dt}={V}_{0}\,\frac{d{c}_{{\rm{a}}}(t)}{dt}$$

and

5 $$\frac{d{m}_{2}(t)}{dt}={K}_{1}\,{c}_{{\rm{a}}}(t)-{k}_{2}^{{\prime} }\,{m}_{2}(t),$$

where the total tracer quantities in the vascular system (both free and bound to plasma proteins), *m*_1_, are distributed in the vascular volume, *V*_0_, and *c*_a_(*t*) reflects the concentration in that volume. Although, *m*_1_ reflects the total quantity of ligand in plasma, only the free amount is exchangable across the BBB (Fig. 1A).

The term *m*_2_ refers to tracer in brain tissue that reflects the clearance *K*_1_ from the circulation and the fractional clearance *k*^′^_2_ from the tissue. Linearized solutions yield the constants *K*_1_ and ${k}_{2}^{{\prime} }$, as previously summarized by Nahimi *et al*.^12^ and Phan *et al*.^16^. Briefly, at steady-state, *V*_T_ equals the ratio ${K}_{1}/{k}_{2}^{{\prime} }$ where *K*_1_ is a function of blood flow, *F*, free plasma fraction, *f*_P_ , and the permeability- surface area of the vascular bed, *PS*,

6 $${K}_{1}={f}_{{\rm{P}}}\,F\,(1-{e}^{-PS/({f}_{{\rm{P}}}F)}),$$

and $${k}_{2}^{{\prime} }$$ is,

7 $${k}_{2}^{{\prime} }=\frac{{k}_{2}}{1+B{P}_{{\rm{ND}}}}=\frac{{K}_{1}}{{V}_{{\rm{ND}}}\,\mathrm{(1}+B{P}_{{\rm{ND}}})}$$

where *k*_2_ is the rate of efflux from *V*_ND_, and *BP*_ND_ is the binding potential. Therefore, at steady-state, the values of *K*_1_ (eq. 6) and ${k}_{2}^{{\prime} }$ (eq. 7) yield,

8 $${k}_{2}={k}_{2}^{{\prime} }\,\mathrm{(1}+B{P}_{{\rm{ND}}})=\frac{{K}_{1}}{{V}_{{\rm{ND}}}}.$$

At steady-state, the *K*_1_/${k}_{2}^{{\prime} }$ ratio defines the total volume of distribution in tissue (*V*_T_) as determined by the total concentration in brain relative to plasma,

9 $${V}_{{\rm{T}}}=\frac{{K}_{1}}{{k}_{2}^{{\prime} }}$$

Equations 4 and 5 together define the exchange of tracer in the brain tissue as a whole,

10 $$\frac{dm(t)}{dt}=\frac{d{m}_{1}(t)}{dt}+\frac{d{m}_{2}(t)}{dt}={V}_{0}\,\frac{d{c}_{{\rm{a}}}(t)}{dt}+{K}_{1}\,{c}_{{\rm{a}}}(t)-{k}_{2}^{{\prime} }\,{m}_{2}(t)$$

from which the total quantity of tracer, *m*, is the sum of the solutions to the differential equations,

11 $$m(T)={m}_{1}(T)+{m}_{2}(T)={V}_{0}\,{c}_{a}(T)+{K}_{1}\,{\int }_{0}^{T}\,{c}_{{\rm{a}}}(t)\,dt-{k}_{2}^{{\prime} }\,{\int }_{0}^{T}\,{m}_{2}(t)\,dt$$

As *m*_2_, is unknown, eq. 11 is solved by substitution of the expression, *m*(1 − *m*_1_/*m*),

12 $$m(T)={V}_{0}\,{c}_{a}(T)+{K}_{1}\,{\int }_{0}^{T}{c}_{{\rm{a}}}(t)\,dt-{k}_{2}^{\prime} \,{\int }_{0}^{T}m(t)\,(1-\frac{{m}_{1}(t)}{m(t)})\,dt$$

but when steady-state is approached, the *m*_1_/*m* ratio becomes negligible and eq. 12 reduces to,

13 $$m(T)={K}_{1}\,{\int }_{0}^{T}\,{c}_{{\rm{a}}}(t)\,dt-{k}_{2}^{{\prime} }\,{\int }_{0}^{T}\,m(t)\,dt$$

#### Volumes of distribution from linearized graphical analysis of tracer kinetics

The second step is the choice of any one of several graphical solutions to eq. 13 based on dynamic measurements of any two of three kinetic variables that include the apparent volume of distribution, *V*_app_, [ml cm^−3^]

14 $${V}_{{\rm{app}}}(T)=\frac{{\int }_{0}^{T}\,m(t)\,dt}{{\int }_{0}^{T}\,{c}_{{\rm{a}}}(t)\,dt},$$

the apparent clearance of radioligand from plasma to brain, *K*_app_, [ml cm^−3^ min^−1^],

15 $${K}_{{\rm{app}}}(T)=\frac{m(T)}{{\int }_{0}^{T}\,{c}_{{\rm{a}}}(t)\,dt},$$

and the apparent residence time in the brain [min],

16 $${\rm{\Theta }}(T)=\frac{{\int }_{0}^{T}\,m(t)\,dt}{m(T)}.$$

Six linearizations previously derived to obtain estimates of *K*_1_ and ${k}_{2}^{{\prime} }$ from steady-state solutions of eq. 13 used combinations of two out of the three dynamic variables (eqs 14, 15 and 16), including two regressions with negative slopes (‘N1’ and ‘N2’), and four regressions with positive slopes (‘P1-P4’). The ‘N1’ graph plotted the apparent clearance as a function of the apparent volume of distribution as derived by Gjedde *et al*.^51^ and Cumming *et al*.^52^ by dividing eq. 13 by ${\int }_{0}^{T}\,{c}_{{\rm{a}}}(t)dt$,

17 $${\bf{N1}}\,{K}_{{\rm{app}}}(T)={K}_{1}-{k}_{2}^{{\prime} }\,{V}_{{\rm{app}}}(T),$$

where *K*_1_ and ${k}_{2}^{{\prime} }$ represent y-intercept and slope, respectively. The ‘N2’ plot was derived by Gjedde *et al*.^53^ as the mirror of ‘N1’,

18 $${\bf{N2}}\,{V}_{{\rm{app}}}(T)={V}_{{\rm{T}}}-\frac{1}{{k}_{2}^{{\prime} }}\,{K}_{{\rm{app}}}(T),$$

where *V*_T_ is the y-intercept and ${k}_{2}^{{\prime} }$ is the reciprocal value of the slope. Division of eq. 13 by *m*(*T*) and *k*_2_′ yields the ‘P1’ plot with a positive slope of the linear relationship between the reciprocal value of *K*_app_(*T*) and Θ(*T*)^51^,

19 $${\bf{P1}}\,\frac{1}{{K}_{{\rm{app}}}(T)}=\frac{1}{{V}_{{\rm{T}}}}{\rm{\Theta }}(T)+\frac{1}{{K}_{1}},$$

where *V*_T_ is the reciprocal value of the slope, and *K*_1_ is the reciprocal value of the y-intercept. Logan and colleagues^54^ derived the ‘P2’ plot as the mirror of ‘P1’,

20 $${\bf{P2}}\,{\rm{\Theta }}(T)={V}_{{\rm{T}}}\frac{1}{{K}_{{\rm{app}}}(T)}-\frac{1}{{k}_{2}^{{\prime} }},$$

where *V*_T_ is the slope of the line. The ‘P3’ plot was introduced by Reith *et al*.^55^ by division of eq. 13 with ${\int }_{0}^{T}\,m(t)\,dt$,

21 $${\bf{P}}{\bf{3}}\,\frac{1}{{\rm{\Theta }}({\rm{T}})}={K}_{1}\frac{1}{{V}_{{\rm{a}}{\rm{p}}{\rm{p}}}(T)}-{k}_{2}^{{\prime} },$$

where *K*_1_ is the slope and ${k}_{2}^{{\prime} }$ the y-intercept. Nahimi *et al*.^12^ described the mirror version, ‘P4’,

22 $${\bf{P4}}\,\frac{1}{{V}_{{\rm{app}}}(T)}=\frac{1}{{K}_{1}}\frac{1}{{\rm{\Theta }}(T)}+\frac{1}{{V}_{{\rm{T}}}}$$

where *K*_1_ is the reciprocal of the slope and *V*_T_ is the reciprocal of the y-intercept. All six plots yielded similar estimates of *K*_1_, ${k}_{2}^{{\prime} }$, and *V*_T_ when the parameters were estimated in the same time frames at steady-state. We applied all six plots because they have different sensitivities to linearity. We note that the Logan plot (‘P2’) and it’s mirror version, ‘P1’ plot, readily but deceptively approached linear relationships that remained linear throughout acquisition time, whereas the alternative plots (‘N1’, ‘N2’, ‘P3’, ‘P4’) lost linearity when steady-state was disrupted (Supplemental Fig. S2). Because the value of *V*_T_ strictly is defined only at steady-state and because the later analysis steps are sensitive to the accuracy of values of *V*_T_, we applied all six plots to know when steady-state is present. The time frames of steady-state were objectively chosen based on the goodness of fit from iterative analysis (as described in details in Data Analysis).

#### Reference Volume of Distribution from Inhibition Plots

The third step is to estimate the volume of distribution of non-displaceable tracer, *V*_ND_, most simply from a brain region with no specific binding of the ligand. If no such region exists, *V*_ND_ can be estimated by pharmacological blocking of specific binding, graphically solved with one of three plots, the so-called Inhibition, Saturation, and Occupancy plots. The Saturation Plot was first derived by Lassen and colleagues^56^,

23 $${V}_{{\rm{T}}({\rm{b}})}=\frac{1}{s}\,{\rm{\Delta }}{V}_{{\rm{T}}}+{V}_{{\rm{ND}}},$$

where Δ*V*_T_, the difference of distribution volumes between baseline and response to pharmacological challenge, is plotted as a function of the volume of distribution at baseline, *V*_T(b)_. The symbol *s* denotes the degree of saturation as the fraction of occupied receptors, and *V*_ND_ is the estimate of the y-intercept. Cunningham *et al*.^57^ derived the Occupancy Plot as the mirror of the Saturation plot,

24 $${\rm{\Delta }}{V}_{{\rm{T}}}=s\,{V}_{{\rm{T}}({\rm{b}})}-s\,{V}_{{\rm{ND}}}.$$

while the Inhibition Plot previously was introduced by Gjedde and Wong^58^ and Gjedde *et al*.^53^, with the distribution volume in the challenge condition *V*_T(i)_ plotted directly as a function of *V*_T(b)_,

25 $${V}_{{\rm{T}}({\rm{i}})}=\mathrm{(1}-s)\,{V}_{{\rm{T}}({\rm{b}})}+s\,{V}_{{\rm{ND}}},$$

where *V*_ND_ is the intercept of the Inhibition Plot with the line of identity.

The three plots were derived from a common expression of the relative receptor availability at inhibition, *BP*_ND(i)_, and baseline, *BP*_ND(b)_,

26 $$1-s=\frac{B{P}_{{\rm{ND}}({\rm{i}})}}{B{P}_{{\rm{ND}}({\rm{b}})}},$$

where 1 − *s* denotes the fraction of available receptors in the presence of a competitor. The *BP*_ND_ equals,

27 $$B{P}_{{\rm{ND}}({\rm{i}})}=\frac{{V}_{{\rm{T}}({\rm{i}})}-{V}_{{\rm{ND}}({\rm{i}})}}{{V}_{{\rm{ND}}({\rm{i}})}},$$

and

28 $$B{P}_{{\rm{ND}}({\rm{b}})}=\frac{{V}_{{\rm{T}}({\rm{b}})}-{V}_{{\rm{ND}}({\rm{b}})}}{{V}_{{\rm{ND}}({\rm{b}})}},$$

where *V*_ND(i)_ and *V*_ND(b)_ denote the distribution volumes of non-displaceable tracer at inhibition and baseline, respectively. When the volumes are constant at the two conditions, (*V*_ND(i)_ = *V*_ND(b)_ = *V*_ND_), eqs 26, 27 and 28 yield the expression of relative receptor availability in terms of volumes of distribution,

29 $$1-s=\frac{{V}_{{\rm{T}}({\rm{i}})}-{V}_{{\rm{ND}}}}{{V}_{{\rm{T}}({\rm{b}})}-{V}_{{\rm{ND}}}},$$

where rearrangement of eq. 29 yields the Inhibition Plot (eq. 25), Saturation (eq. 23), and Occupancy (eq. 24) plots.

A requirement of application of any plot is that *f*_P_ , the free fraction of radioligand in plasma, must remain unchanged at baseline and challenge. At steady-state, the *V*_ND_ value is the ratio of the total concentrations of radioligand in blood, *C*_a_, and brain, *C*_ND_,

30 $${V}_{{\rm{ND}}}={V}_{{\rm{D}}}\,\frac{{C}_{{\rm{ND}}}}{{C}_{{\rm{a}}}}$$

where *V*_D_ is the volume in which *C*_ND_ is the concentration. The concentrations *C*_a_ and *C*_ND_ also are the concentrations of freely dissolved ligands in plasma *C*_FP_ and tissue water, *C*_FT_,

31 $${C}_{{\rm{FP}}}={f}_{{\rm{P}}}\,{C}_{{\rm{a}}},$$

and

32 $${C}_{{\rm{FT}}}={f}_{{\rm{FT}}}\,{C}_{{\rm{ND}}}.$$

such that the combination of eqs 30, 31 and 32 yields,

33 $${V}_{{\rm{ND}}}={V}_{{\rm{D}}}\,\frac{{f}_{{\rm{P}}}}{{f}_{{\rm{ND}}}}\frac{{C}_{{\rm{FT}}}}{{C}_{{\rm{FP}}}}$$

where *V*_ND_ is a product of a physical volume, *V*_*D*_, and two unitless ratios. The *f*_P_ to *f*_ND_ ratio depends on the non-specific binding in plasma and brain tissue, while the *C*_FT_ to *C*_FP_ ratio depends on properties of the blood-brain barrier (BBB). Assuming that the radioligand enters the brain tissue by passive diffusion, and free concentrations at steady-state the of ligand across BBB are equal, eq. 33 reduces to,

34 $${V}_{{\rm{ND}}}={V}_{{\rm{D}}}\,\frac{{f}_{{\rm{P}}}}{{f}_{{\rm{ND}}}}.$$

such that a rise of *f*_P_ would change the magnitude of *V*_ND_.

#### Variable reference volumes of distribution from Extended Inhibition Plot

The fourth step is the correction for a change of the reference volume of distribution when plasma free fractions, *f*_P_ , have changed in response to pharmacologcial challenge. In this case, eq. 29 can be applied in its unreduced form,

35 $$1-s=(\frac{{V}_{{\rm{T}}({\rm{i}})}-{V}_{{\rm{ND}}({\rm{i}})}}{{V}_{{\rm{T}}({\rm{b}})}-{V}_{{\rm{ND}}({\rm{b}})}})\,[\frac{{V}_{{\rm{ND}}({\rm{b}})}}{{V}_{{\rm{ND}}({\rm{i}})}}],$$

where *V*_ND(b)_ and *V*_ND(i)_ are the magnitudes at baseline and at inhibition. Isolation of *V*_T(i)_ in eq. 35 yields the Extended Inhibition Plot,

36 $${V}_{{\rm{T}}({\rm{i}})}=\mathrm{(1}-s)\,[\frac{{V}_{{\rm{ND}}({\rm{i}})}}{{V}_{{\rm{ND}}({\rm{b}})}}]\,{V}_{{\rm{T}}({\rm{b}})}+s\,{V}_{{\rm{ND}}({\rm{i}})},$$

takes the changes of *f*_P_ and *V*_ND_ into account. The value of *V*_ND(b)_ must be known to solve for *V*_ND(i)_ in eq. 36, for example with reference to a pharmacological challenge that affects the receptor binding without altering *f*_P_ , by modulating the release or depletion of endogenous transmitter. In contrast, challenge with a unlabeled ligand may raise *f*_P_ , when the challenge with unlabeled ligand displaces ligand bound to plasma proteins. Challenge with unlabeled yohimbine may reduce the protein binding of the radioligand when as much as 82% of labeled yohimbine is bound to plasma proteins^22^.

#### Calculation of binding potentials from total and reference volumes

The fifth step is the calculation of binding potentials from volumes of distribution. The binding potential, *BP*_ND_, is the ratio of specifically bound to non-displaceable ligand quantities in the tissue^20,21^. The *BP*_ND_ is determined from the total volume of distribution in the tissue, *V*_T_, and the volume of distribution of non-displaceable ligand, *V*_ND_, determined at the respective conditions of variable occupancy,

37 $$B{P}_{{\rm{ND}}}=\frac{{V}_{{\rm{T}}}-{V}_{{\rm{ND}}}}{{V}_{{\rm{ND}}}}.$$

where *V*_ND_ is obtained from a reference region devoid of specific receptors. In the case of yohimbine, *α*_2_ adrenoceptors are widely distributed in the brain, with no suitable reference region.

#### Determination of *B*_max_ and *K*_D_ estimates from Eadie-Hofstee Plot

The sixth and final step was the graphical solution to the Eadie-Hofstee Plot of eq. (1) (Eadie 1952, Hofstee 1952), where *B* is the bound quantity and *BP*_ND_ is the binding potential of ligands. We calculated the bound quantity from the relationship described by eq. 2.

### Animals

Animal experiments were conducted according to the protocol approved by the Danish Animal Experiments Inspectorate in compliance with the law regulated by the Danish Ministry of Food, Agriculture and Fisheries. Five female Sprague Dawley rats (weighing 250–300 g) were housed two per cage with access to food and water ad libitum under 12/12 hours light/dark cycle conditions. The animals had two consecutive 90-minute PET acquisitions on the same day. Anesthesia was induced in a chamber with 5% isoflurane, maintained by mask with 2% isoflurane until the end of PET recording. To raise the delivery of yohimbine to the brain by limiting the reaction of yohimbine with p-glycoprotein at the BBB^59^, we gave animals cyclosporine i.v. 50 mg/kg 30 minutes before the baseline PET. With a half-life longer than 10 hours at doses exceeding 30 mg/kg^60^, only one dose was needed. We administered unlabeled yohimbine 5–10 min before the second acquisition at an IV dose of 0.3 mg/kg.

### PET acquisitions and image processing

Animals were imaged in a tomograph designed for rodents (microPET R4, CTI Concorde Microsystems, Knoxville, TN, USA), with a spatial resolution at the center of the field of view at 3.69 mm^3^ ^61^. PET images were processed with MINC software of McConnell Brain Imaging Centre, Montreal Neurological Institute, McGill University. We reconstructed the photon attenuation correction of dynamic recordings by 3D-filtered back projection, resulting in a 128 × 128 × 63 matrix. Summed emission recordings were coregistered to a digital MRI atlas of the rat brain, using the program Register of MINC software. The dynamic emission recordings were resampled to the same 3D-space of the MRI rat brain atlas. We used masks of volumes of interest (VOIs) developed by Schiffer *et al*.^62^, which we resampled to MRI space. The masks were then used to extract time-activity curves from the resampled dynamic data sets.

### Data analysis

We developed the user-friendly, open-source graphical user interface Kinetic Windows (KiWi) in MATLAB to aid robust and effective kinetic analysis. KiWi enables users to import time-activity curves, to plot dynamic PET parameters as functions of time (eqs 14–16), and ultimately to simultaneously plot data with the N1–N2 or P1–P4 plots. The uptake phase with the optimal dynamic data fit is objectively chosen in KiWi, identified as the frames with the r-squared precision closest to unity. The software is available on Github^63^ and MATLAB file exchange^64^. Time-activity curves for each region were analyzed together with the corresponding plasma activity curve as input. Here, we chose estimates of *V*_T_ obtained graphically from N2 plot (eq. 18) derived by Gjedde *et al*.^53^. Parametric maps of *BP*_ND_ were constructed by means of eqs 27 and 28.

### Plasma Ultrafiltration

To test the change of *f*_P_ incurred by unlabeled yohimbine challenge relative to baseline, plasma ultrafiltration was performed as previously described by Gandelman *et al*.^65^ and plasma free fractions quantified both *ex vivo* and *in vitro* (the method is illustrated in Fig. 2D left). In the *ex vivo* experiment, a separate group of six female Sprague Dawley rats were subjected to plasma ultrafiltration and treated identically to animals that underwent PET acquisition. We administered cyclosporine (50 mg/kg) i.v., and we sampled arterial blood by catheter in the femoral artery. Samples from the baseline condition were collected 30 min after the cyclosporine treatment. To obtain blood from the unlabeled ligand challenge condition, we administered unlabeled yohimbine (0.3 mg/kg i.v.) and collected blood samples after 30 min. We centrifuged the blood samples in heparinized tubes for 20 min at 4 °C to generate plasma and stored the samples at −50 °C.

In the *in vitro* experiments, blood was drawn and yohimbine or amphetamine were added to the plasma samples later. The amounts of yohimbine and amphetamine added to the samples were calculated from the linear relationship between blood volume and body weight in rats^66^. The calculation assumed that plasma comprises 50% of the blood volume in rats and that the drug initially is distributed throughout plasma. The calculated amounts of drug were added to individual plasma samples. Thus 5 *μ*g corresponded to a low yohimbine dose of 0.3 mg/kg and 25 *μ*g to a higher dose of 1.5 mg/kg.

To measure *f*_P_ , we used the Centrifree centrifugal filter device (Millipore, Bedford, MA) with a molecular cutoff of 30 kDa for all samples. This pore size was chosen to ensure filtration of the yohimbine fraction bound to albumin with a molecular weight of approximately 65 kD in rats^67^. On the day of *f*_P_ determination, the plasma samples were thawed in room temperature in 20 min. Each sample unit of 1000 *μ*l plasma was spiked with 50 *μ*l [^11^C]yohimbine. After 10 minutes incubation at room temperature, aliquots of 50 *μ*l of this solution were used to measure the total activity (unfiltered plasma). For triplicate measurements of the filtered plasma, the remaining volume was divided into three ultrafiltration devices and centrifuged for 20 min using a centrifuge with a fixed angle rotor at 1000 g. After filtration, we removed aliquots of 50 *μ*l to assess the activity in protein-free plasma. The activity of filtered plasma was determined in triplicates. All samples were counted in a gamma device (Packard Cobra Gamma Counter, Model D5003). Non-specific binding of the tracer to the ultrafiltration device was recovered by performing the same procedure on a phosphate-buffered solution. The correction factor, *r*, was determined as the ratio of the activity in the phosphate buffer before filtration, *A*_buffer(total)_, to the filtered buffer, *A*_buffer(ultrafiltrate)_,

38 $$r=\frac{{A}_{{\rm{buffer}}({\rm{total}})}}{{A}_{{\rm{buffer}}({\rm{ultrafiltrate}})}},$$

from which the plasma-free fraction, *f*_P_ , was calculated as the ratio of the activity of the filtered plasma, *A*_plasma(ultrafiltrate)_, to the unfiltered plasma, *A*_plama(total)_, multiplied by the correction factor, *r*, for recovery,

39 $${f}_{{\rm{P}}}=r\,\frac{{A}_{{\rm{plasma}}({\rm{ultrafiltrate}})}}{{A}_{{\rm{plasma}}({\rm{total}})}},$$

### Statistics

To determine *f*_P_ elevation in response to unlabeled yohimbine challenge, we applied a non-parametric two-tailed test for the *ex vivo* assay. For the *in vitro* assay, we applied two-way ANOVA followed by Tukey’s post-hoc analysis. To examine whether yohimbine challenge changed *V*_T_ and *BP*_ND_ compared to baseline, two-way ANOVA followed and Tukey’s post-hoc analysis was applied. For all tests, a probability of less than 0.05 was considered significant. Statistical analyses were carried out in GraphPad Prism v7.

## Electronic supplementary material

Supplementary material
