# Author Correction: Radioligand binding analysis of *α*_2_ adrenoceptors with [^11^C]yohimbine in brain in vivo: Extended Inhibition Plot correction for plasma protein binding

**DOI:** 10.1038/s41598-018-24170-z

**Published:** 2018-04-16

**Authors:** Jenny-Ann Phan, Anne M. Landau, Steen Jakobsen, Dean F. Wong, Albert Gjedde

**Affiliations:** 1 Department of Biomedicine, Aarhus University, Aarhus, 8000 Denmark; 2 Department of Nuclear Medicine and PET centre, Aarhus University Hospital, Aarhus, 8000 Denmark; 3 Department of Radiology & Radiological Science, Johns Hopkins University Hospital, Baltimore, 21231 USA; 4 Translational Neuropsychiatry Unit, Aarhus University Hospital, Aarhus, 8240 Denmark; 5 Radiology, Psychiatry, Neuroscience, Neurology, Environmental Health Sciences, Johns Hopkins University, Johns Hopkins Medical Institutions, JHOC Bldg room 3245, 601 N. Caroline St., Baltimore, MD 21287 USA; 6 Department of Neuroscience, University of Copenhagen, Copenhagen, 2200 Denmark; 7 Department of Neurology and Neurosurgery, McGill University, Montréal, Québec, Canada; 8 Neurosciences Research Center, Tabriz University of Medical Science, Tabriz, Iran; 9 Department of Nuclear Medicine, Odense University Hospital, Odense, 5230 Denmark; 10 Department of Clinical Medicine, University of Southern Denmark, Odense, 5230 Denmark

Correction to: *Scientific Reports* 10.1038/s41598-017-16020-1, published online 22 November 2017

Dean F. Wong was omitted from the author list in the original version of this Article. This has been corrected in the PDF and HTML versions of the Article, and in the accompanying Supplementary Material file.

The Acknowledgements section now reads:

“The authors thank Professor Doris Doudet for constructive scientific discussions and suggestions. We thank Mette Simonsen for technical assistance with PET acquisitions and blood sampling. Jenny-Ann Phan is a recipient of an MD-PhD fellowship from Aarhus University. The experiments were funded by Department of Nuclear Medicine and PET Centre, Aarhus University Hospital and by grants from the Council of Independent Research, Denmark. All authors declare no conflicts of interest, including relevant financial interests, activities, relationships, and affiliations. DFW acknowledges PHS NIH grant 2 R01 MH107197.”

The Author Contributions section now reads:

“J.A.P. designed, planned, and performed the experiments, analyzed data, and wrote scripts for the graphical user interface in MATLAB. A.M.L. contributed to study design, planning and assistance with image quantification. S.J. was responsible of radioligand production. D.F.W contributed to the content of the paper and interpretation of results of the study. A.G. conceived the study, designed the kinetic analyses, and supervised the work throughout the study. J.A.P. and A.G. wrote the manuscript.”

Additionally, this Article contained a typographical error in the Methods section.

“As *m*_2_, is unknown, eq. 11 is solved by substition of the expression, *m*(1 − *m*_1_/*m*),”

now reads:

“As *m*_2_, is unknown, eq. 11 is solved by substitution of the expression, *m*(1 − *m*_1_/*m*),”

Finally, this Article contained errors in Equation 12.

$$m(T)={V}_{0}{c}_{a}(T)+{K}_{1}{\int }_{0}^{T}{c}_{a}(t){dt}-{k}_{2}^{^{\prime} }{\int }_{0}^{T}m(t)(\frac{1-{m}_{1}(t)}{m(t)}){dt}$$

now reads:

$$m(T)={V}_{0}{c}_{a}(T)+{K}_{1}{\int }_{0}^{T}{c}_{a}(t){dt}-{k}_{2}^{^{\prime} }{\int }_{0}^{T}m(t)(1-\frac{{m}_{1}(t)}{m(t)}){dt}$$

These errors have now been corrected in the PDF and HTML versions of the Article, and in the accompanying Supplementary Information file.

