# Supplementary material for: Radioligand binding analysis of α2 adrenoceptors with [11C]yohimbine in brain in vivo: Extended Inhibition Plot correction for plasma protein binding
Source: Sci Rep. 2017 Nov 22;7:15979. doi: 10.1038/s41598-017-16020-1 (PMC5700124; doi:10.1038/s41598-017-16020-1)
Supplement: Supplementary file 1 — Supplementary material [file 41598_2017_16020_MOESM1_ESM.pdf]

Radioligand binding analysis of α_2 adrenoceptors with [^{11}C]yohimbine in brain in vivo: Extended Inhibition Plot correction for plasma protein binding

Jenny-Ann Phan, Anne M. Landau, Steen Jakobsen, Dean F. Wong, Albert Gjedde

Figure S1. Representative Dynamic Variables

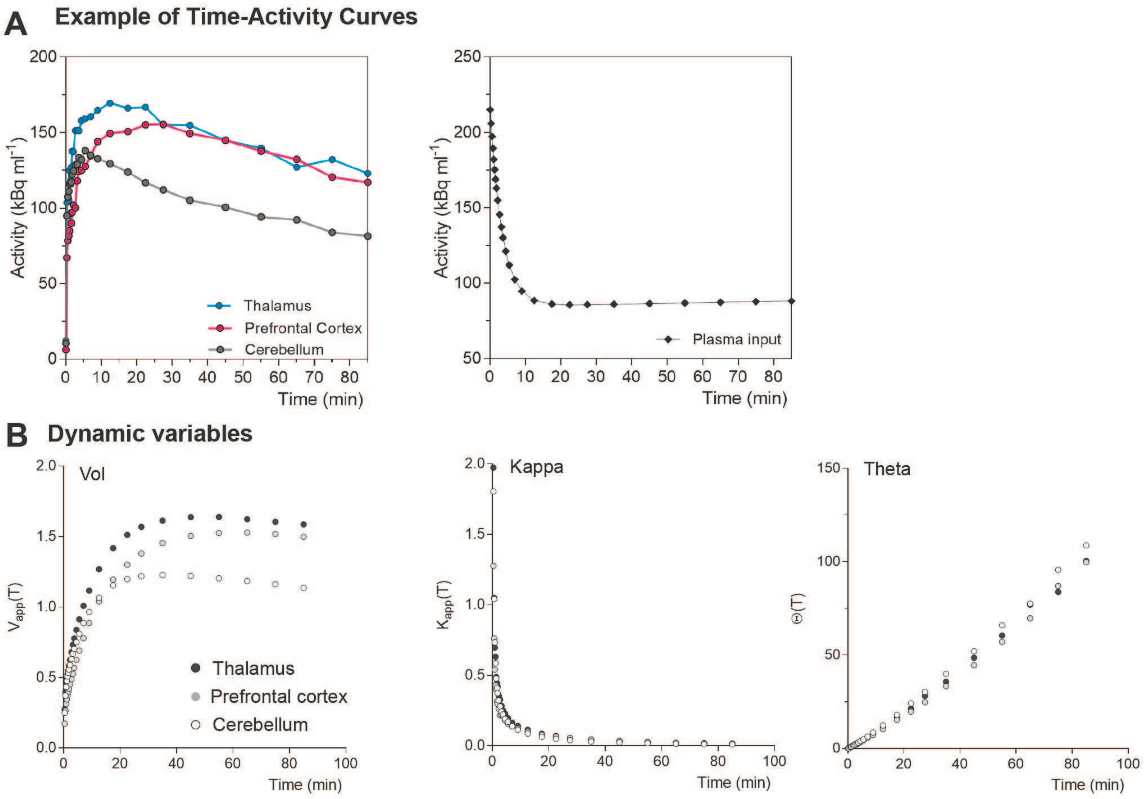

Figure S2. Linearized Graphical Models and Goodness of Fit

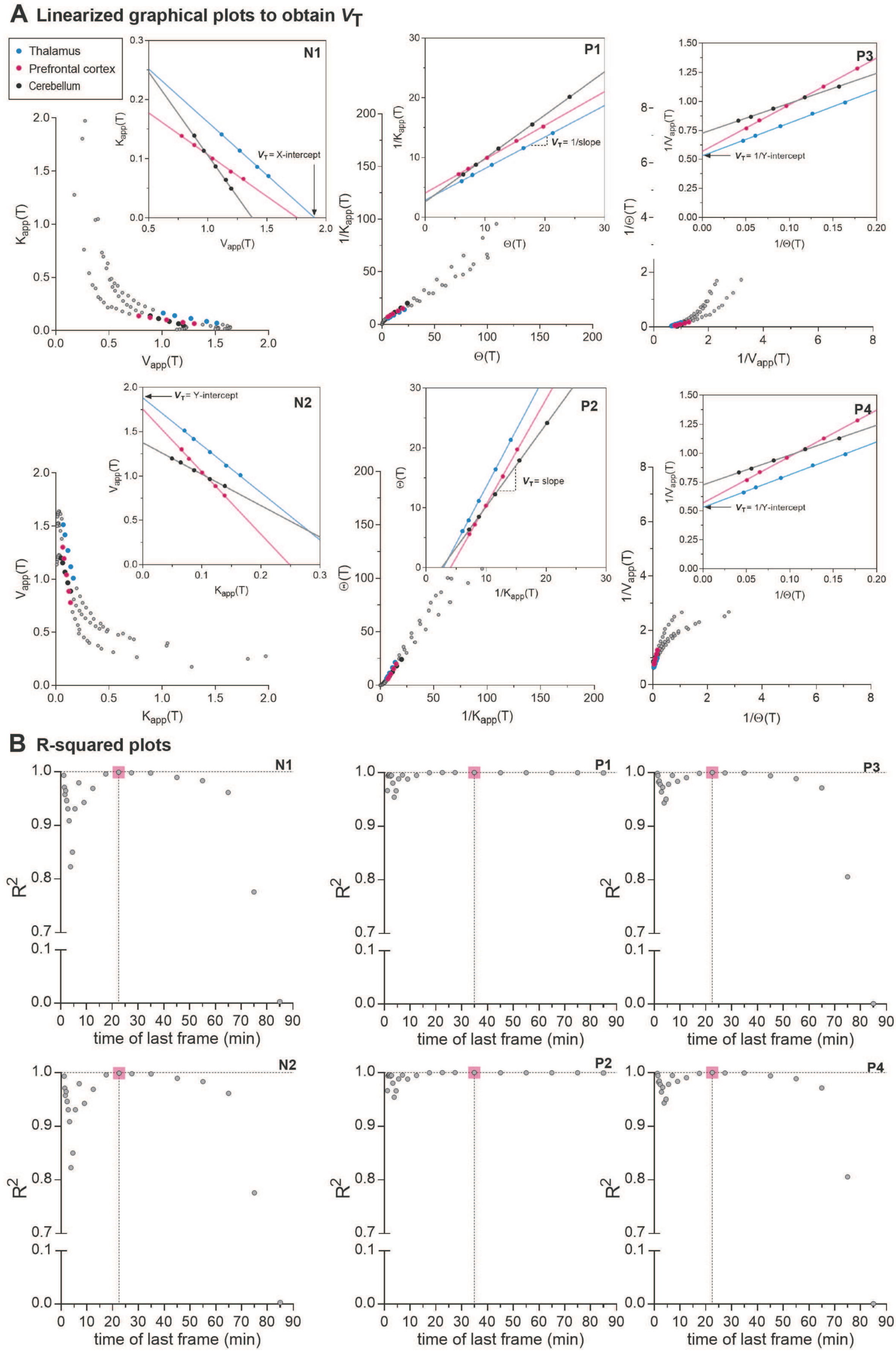

Legend to Figure S1. Dynamic Variables and Linearized Graphical Models Representative time-activity curves in brain and plasma are presented in (**A**) to demonstrate the analysis methods applied in this study. The three dynamic variables (defined by eqs. 14, 15 and 16 in Methods) are calculated from the representative data set and presented in **B**. These variables and their reciprocal counterparts are applied in the graphical linearizations to solve K_1, k_2' and V_T in the following **Supplemental Figure S2**.

Legend to Figure S2. Linearized Graphical Models and Goodness of Fit Panel **A** shows the six linearized plots of dynamic data from thalamus, prefrontal cortex and cerebellum. The inserts in **A** correspond to data fitted in the time interval of 7-22.5 min. This interval was identified as the period with the greatest R^2 that approximated 1, and this period is also held as the steady-state period. The time interval of steady-state was objectively identified by iterative linearization of five acquisition frames using our custom-written software, KiWi, in MATLAB. Each plot has different graphical properties, which are indicated in the inserts in **B**. For example V_T is the X-intercept on N1 plot, Y-intercept on N2 plot and the slope on P2 plot (also known as Logan Plot). Whereas, V_T is obtained as the reciprocal variables on the other plots, such as the reciprocal slope on P1, reciprocal X-intercept on P3, and reciprocal Y-intercept on P4.

The calculation of V_ND in the later analysis is highly sensitive to the accuracy of V_T, therefore it is crucial to confirm that steady-state is present in the time period of V_T estimation. Panel **B** shows R^2 plots derived from iterative fitting of dynamic data in prefrontal cortex to evaluate the accuracy of the fits. Each R^2 value represents the goodness of fit of each linearization. The pink square indicates the fit period with the highest R^2 value on respective plots. This revealed that there was a consensus of steady-state was present at 7-22.5 min for four out of the six plots (N1, N2, P3, P4 plots). Interestingly, P1 and P2 show that all fits from 20 min and onward have R^2 values that all approximated 1. The period of best fit according to P1 and P2 were found at 12.5-35 min. However, the difference of R^2 estimates in this period in comparison with the other acquisition periods was very small because the difference was found in the fourth decimal. Notably, N1, N2, P3 and P4 plots revealed that R^2 values started to drop markedly from 45 min until the end of the acquisition, but this information was missing on P1 and P2 plots. By plotting all six plots, we were able to fit V_T at steady-state, which would not have been possible by using only Logan plot.
